# Impact of Modern Low Dose Involved Site Radiation Therapy on Normal Tissue Toxicity in Cervicothoracic Non-Hodgkin Lymphomas: A Biophysical Study

**DOI:** 10.3390/cancers15245712

**Published:** 2023-12-05

**Authors:** Julian Roers, Daniel Rolf, Andrea Baehr, Christoph Pöttgen, Martina Stickan-Verfürth, Jan Siats, Dominik A. Hering, Christos Moustakis, Maximilian Grohmann, Michael Oertel, Uwe Haverkamp, Martin Stuschke, Beate Timmermann, Hans T. Eich, Gabriele Reinartz

**Affiliations:** 1 Department of Radiation Oncology, University Hospital of Münster, West German Cancer Center (WTZ) Network Partner Site, Albert-Schweitzer-Campus 1, 48149 Münster, Germany; 2 Department of Radiation Oncology, University Hospital of Hamburg-Eppendorf, Martinistraße 52, 20246 Hamburg, Germany; 3 Department of Radiation Oncology, University Hospital of Essen, West German Cancer Center (WTZ), Hufelandstraße 55, 45147 Essen, Germany; 4 Department of Particle Therapy, University Hospital of Essen, West German Proton Therapy Center Essen (WPE), West German Cancer Center (WTZ), Am Mühlenbach 1, 45147 Essen, Germany; 5 Department of Radiation Oncology, University Hospital of Leipzig, Stephanstraße 9a, 04103 Leipzig, Germany

**Keywords:** non-Hodgkin lymphoma, involved site radiation therapy, normal tissue toxicity, Lyman–Kutcher–Burman model, reduced radiation doses

## Abstract

**Simple Summary:**

The effects of reduced prescription doses on normal tissue toxicity in the treatment of non-Hodgkin lymphomas of the neck/thorax region remain unclear. Acute side effects, which already occur at low organ doses, can only be described to a limited extent by the models given in the literature. Nonetheless, the knowledge about these dose–response relationships is particularly important to predict an acute response in normal tissue adequately. Considering a robust normal tissue toxicity model, dose prescriptions and treatment plans can be optimized to increase the treatment quality and, thus, the outcome for the patient.

**Abstract:**

This biophysical study aimed to determine fitting parameters for the Lyman–Kutcher–Burman (LKB) dose–response model for normal tissue complication probability (NTCP) calculations of acute side effects and to investigate the impact of reduced radiation doses on the probability of their occurrence in supradiaphragmatic non-Hodgkin lymphoma (NHL) irradiation. A cohort of 114 patients with NHL in the cervicothoracic region, treated between 2015 and 2021 at the University Hospitals of Münster, Hamburg, and Essen, with involved site radiation therapy (ISRT), were included. Among them, 68 patients with aggressive NHL (a-NHL) received consolidative radiation therapy with 24–54 Gy following (R-)CHOP chemotherapy. Additionally, 46 patients with indolent NHL (i-NHL) underwent radiotherapy with 22.5–45.0 Gy. Two treatment plans were prospectively created for each patient (a-NHL: 30.0/40.0 Gy; i-NHL: 24.0/30.0 Gy). NTCP were then calculated using the optimized LKB model. The adapted dose–response models properly predicted the patient’s probability of developing acute side effects when receiving doses ≤ 50 Gy. In addition, it was shown that reduced radiation doses can influence the NTCP of acute side effects depending on the aggressiveness of NHL significantly. This study provided a foundation to prospectively assess the probability of adverse side effects among today’s reduced radiation doses in the treatment of NHL.

## 1. Introduction

Non-Hodgkin lymphomas (NHL) are a heterogeneous group of malignancies originating from lymphoid tissues and represent a significant health burden worldwide. The management of NHL often involves a multimodal approach, with radiotherapy playing a crucial role in achieving local control and improving patient outcomes [1]. 

In recent years, there has been a growing interest in optimizing radiation therapy strategies to minimize treatment-related toxicities while maintaining effective disease control. Involved site radiation therapy (ISRT) has emerged as a standard treatment approach for supradiaphragmatic NHL, aiming to deliver radiation treatment precisely to the involved lymphatic regions while sparing adjacent normal tissues. Traditionally, radiation doses have been prescribed based on historical guidelines, which may result in acute to long-term side effects. Multiple studies have evaluated the reduction in treatment volume and prescribed dose as well as the adoption of modern conformal techniques, in order to limit treatment-induced side-effects [2]. In 2011, a large, randomized trial confirmed the equivalence in terms of response rate and local control of 24 Gy for indolent NHL and 30 Gy in 2 Gy fractions for aggressive NHL, compared with the doses previously considered as standard (40–45 Gy) [3]. Furthermore, the reduction in treatment volume from mantle field to involved-field radiotherapy first and further to involved-site or -node radiotherapy demonstrated comparable control of disease with reduced irradiation of organs at risk (OAR) [4]. Modern combined treatment for NHL consisted of personalized PET-guided therapy and improved the prognosis of this subset of patients, emphasizing the importance of acute and long-term toxicity [5]. Moreover, with advances in treatment planning techniques, including intensity-modulated radiotherapy (IMRT) and proton beam therapy (PBT), it is now possible to tailor radiation doses to individual patients, potentially reducing toxicity without compromising treatment efficacy [3,6,7,8,9,10,11,12]. 

The evaluation of normal tissue toxicities is essential in assessing the safety and effectiveness of radiation therapy. Radiation-induced toxicity encompasses multiple acute and late side effects, such as radiation pneumonitis, cardiac toxicity, and esophagitis [6,13,14,15,16,17,18,19,20]. The evaluation of normal tissue toxicities is essential in assessing the safety and effectiveness of radiation therapy. Several studies have investigated the effects of field-size and dose prescription on different radiation-induced side effects. For example, Reinartz et al. reported that reducing doses significantly impacted neighbouring organs in gastric lymphoma patients [6]. The use of biophysical dose–response relationships helped to predict adverse events for OAR, particularly for liver, spleen, and bowel toxicity, with corresponding normal tissue complication probability (NTCP) for elevated liver enzymes, low platelet count, and diarrhoea. These findings underscore the substantial impact of field and dose reduction on OAR-dose burden and NTCP during stomach radiation, providing valuable insights for modern radiation techniques in abdominal organ sparing [6]. However, the influence of reduced radiation doses on the extent of NTCP, specifically in the context of NHL of the neck/thorax region, remains unclear [16,21,22,23,24,25]. 

This study aims to investigate the biophysical aspects of reduced radiation doses in the treatment of supradiaphragmatic NHL, focusing on the effects on normal tissue toxicity. By utilizing the probit-based Lyman–Kutcher–Burman (LKB) model for NTCP calculation, optimized model parameters were determined to quantify the relationship between the incidence of radiation-induced acute toxicities and to adequately represent the observed NTCP in the patient cohort [6,13,17,18,19]. With the help of these adapted models, we secondly seek to evaluate the implications of reduced radiation doses on the occurrence of specific toxicities, such as nausea and dysphagia. Understanding the relationship between radiation doses and normal tissue toxicities is crucial for optimizing treatment protocols and improving patient quality of life [15,21,25]. The findings from this study may provide valuable insights into the feasibility of using reduced radiation doses in the management of NHL, particularly in the neck/thorax region. Such knowledge can guide treatment decision making, minimize short- to mid-term complications, and enhance the overall therapeutic outcomes for patients.

## 2. Materials and Methods

**Study design and patient population:** This multicenter study includes a cohort of 114 patients with NHL in the neck/thorax region who underwent treatment between 2015 and 2021 at the University Hospitals of Münster (*i* = 59), Hamburg (*i* = 16), and Essen (*i* = 39). Two patients from Essen were treated with proton beam therapy (PBT) at the West German Proton Therapy Center (WPE). Note that the treatment of NHL in clinical practice is performed with different radiation qualities and techniques. Consequently, PBT is explicitly considered in this work. Patients were selected based on specific inclusion criteria, including NHL diagnosis, involvement of the neck/thorax region, and availability of complete treatment and follow-up data. The median age of the patients was 59 years (range, 19–88). Detailed patient characteristics are summarized in Table 1. 

**Treatment technique and radiation dose regimes:** All patients received ISRT using the IMRT technique or PBT. The treatment planning process involved accurate delineation of target volumes and OAR based on established guidelines [1,7]. IMRT and PBT plans were formulated using optimization goals for both the target and OAR. These plans were designed to align with the dose distribution recommendations of the ICRU Report 83 and the dose constraints to OAR outlined inter alia in the Timmerman tables [26,27,28]. Treatment plans were generated with Varian eclipse™ v15.7, TomoTherapy^®^ planning v.5.1.8, or Raystation^®^ Version 10B and v4.5. The primary objective was to achieve optimal target coverage at the prescribed dose, while concurrently minimizing toxicity. Especially in lymphoma radiotherapy, emphasizing target coverage over minimizing normal tissue complications is crucial due to the lower required doses compared to other cancers. Studies, including those on orbital lymphomas and lung cancers, underscore the importance of maintaining target coverage despite the potential risks to surrounding tissues [22]. The patient cohort was divided into two subgroups based on the histopathological aggressiveness of NHL. The subgroup of aggressive NHL (a-NHL) consisted of 68 patients who received consolidative radiation therapy with doses ranging from 24 Gy to 54 Gy (mean = 40.3 Gy, median = 39.6 Gy) following (R-)CHOP chemotherapy. The subgroup of indolent NHL (i-NHL) included 46 patients who underwent definitive radiation therapy with doses ranging from 22.5 Gy to 45.0 Gy (mean = 34.1 Gy, median = 36.0 Gy).

**Assessment of acute side effects:** Radiogenic acute side effects were evaluated and classified according to the Common Terminology Criteria for Adverse Events (CTCAE) grading system (grades 1–3) [29]. The severity of side effects was assessed during and after the completion of radiation therapy at regular intervals. 

**Calculation of normal tissue complication probabilities (NTCP):** A variety of different mathematical functions exist to describe the relationship between dose and tissue response. The LKB model is often mentioned as the classical dose–response model [16,19,24,30,31]. Its suitability for predicting probabilities of acute side effects was confirmed in previous projects [6]. It can be expressed using only the parameter corresponding to the dose necessary to cause 50% tissue response ${TD}_{50}$; the volume dependency parameter *n,* which quantifies the sensitivity of the irradiated volume of the OAR; and the slope-parameter *m* (often also denoted as $\gamma_{50}=1/(\sqrt{\pi }\cdot m$)), which is the gradient of the dose–response curve at the level of 50% toxicity. The LKB model takes the following form:

(1) $$NTCP\left(D\right)=\frac{1}{\sqrt{2\pi }}\int_{-\infty }^{t(D)}\exp\left(\frac{-{t(D)}^{2}}{2}\right)dt$$

whereas the upper integral limit t is defined as the following:

$$t\left(D\right)=\frac{D-TD_{50}}{m\cdot TD_{50}}$$

For non-uniform volume irradiations ($V_{eff}<1$), the effective volume method introduced by Kutcher and Burmann [19] was applied to transform the tolerance dose for 50% complications $TD_{50}$ for uniform whole organ irradiation into the 50% tolerance dose $TD_{50}(V_{eff})$ for uniform organ irradiation to the effective volume $V_{eff}$:

$$TD_{50}\left(V_{eff}\right)=TD_{50}{V_{eff}}^{-n}$$

where $V_{eff}$ is given by the following:

$$V_{eff}=\sum_{i=1}^{k}v_{i}\cdot {\left(\frac{d_{i}}{d_{ref}}\right)}^{\frac{1}{n}}$$

The cumulative distribution function from Equation (1) can be solved using a probit function, which is related to the error function (erf) [32]:

$$NTCP\left(d_{ref},V_{eff}\right)=\frac{1}{2}\left[1-\mathrm{erf}\left(m^{-1}(1-\frac{d_{ref}}{TD_{50}(V_{eff})})\right)\right]$$

In the LKB model, $d_{ref}$ was taken as the maximum dose to the specific OAR, so that $V_{eff}\leq 1$. In this study, the required model parameters were determined by using the Levenberg–Marquardt algorithm (LMA) to minimize the sum of squared errors between observed and predicted complication probabilities, implemented by hand in the open-source statistical software environment R version 4.2.2 [33]. Note that the volume dependency parameter *n* remained constant in the optimization and only the parameters ${TD}_{50}$ and m were adjusted. 

The patients examined in this study were treated with fractional doses $f_{d}$ in a range from 1.8 to 3 Gy. Therefore, all dose bins $D_{i}$ from the patient-specific dose–volume histograms (DVH), were converted to a standard fractional dose of 2 Gy using the concept of equivalent dose ${EQD}_{2Gy}$ expressed by the linear-quadratic model [34,35]:

$$EQD_{2Gy,i}=d_{i}\left[\frac{\frac{\alpha }{\beta }+f_{d}}{\frac{\alpha }{\beta }+2}\right]$$

The published data of α/β for normal tissue show heterogeneity. Generally, a low α/β indicates a late response in tissue and a high ratio suggests an early response, e.g., in tumor tissue. As an approximation, α/β was set to 3, corresponding to late-responding tissues for all calculations of NTCP [20,36].

A deviation of ≥5% between the observed and predicted NTCP calculated with the existing LKB model caused us to adapt the model parameter. A previous internal work report showed that deviations < 5% are what lead to the fact that the putatively optimized model does not describe the observed dose–response relationship any better than the pre-existing model. Such conditions can be identified with the Brier skill score as described below.

**Statistical methods:** The model fit was checked using a variety of quality criteria. The difference between the actual observed patient NTCP and the predicted complication probability was expressed via Brier score [37]: A Brier score can take any value between 0 and 1—where 0 is the best and 1 is the worst achievable score. The model performance was also verified using the Brier skill score, whereby a positive score indicates that the adapted model provides a more accurate forecast compared to the pre-existing one. To evaluate the quality of fit, a regression analysis was performed by calculating the sum of squared residuals ($R^{2}$, or R-squared) and adjusted $R^{2}$. To clarify, in the case of a perfect fit, $R^{2}=1$. In the worst case, $R^{2}=0$. While R-squared is typically not ideal for assessing the fit of nonlinear regression models like the LKB model, it becomes applicable as a quality criterion when using nonlinear least squares methods such as LMA. In addition, the adjusted $R^{2}$ is used as an indicator to check whether the model is overfitted. The Akaike information criterion divided by the number of data points (AIC/i) was also calculated for completeness.

**Evaluating the effect of reduced radiation doses:** For each patient, two additional IMRT plans were produced by renormalizing the original plans, aiming to simulate reduced radiation doses while preserving plan quality. For the a-NHL subgroup, the plans consisted of doses of 30.0 Gy and 40.0 Gy, while for the i-NHL subgroup doses of 24.0 Gy and 30.0 Gy were used. The differences in NTCP between different treatment plans calculated with the adapted LKB model were analyzed by using a *t*-test for the difference between two means. Significance was considered at α=0.05.

## 3. Results

**Patient characteristics and observed acute side effects:** The cohort consisted of 114 patients with NHL in the neck/thorax region, including 68 patients with aggressive NHL (a-NHL) and 46 patients with indolent NHL (i-NHL). During the course of treatment, radiogenic acute side effects were observed and graded according to the CTCAE criteria. The most common acute toxicities in both the a-NHL and i-NHL subgroups were nausea, esophagitis, and dysphagia. The incidence and severity of acute side effects varied among patients and treatment regimens. The patient outcome data are summarized in Table 1. While the observed NTCP for radiation-induced esophagitis with and without dysphagia and nausea was ≥5%, it was <5% for acute and mid-term side effects associated with radiation pneumonitis, cardiac toxicity, or myelitis. As mentioned before, the NTCP model parameters of such organs were, due to a deviation of <5% between observed and predicted NTCP, not further adapted. 

**Figure 1:**
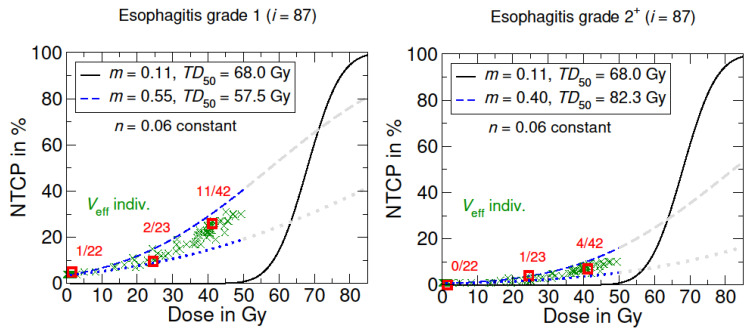
NTCP models for radiation-induced acute side effects to the esophagus. The black curve shows the origin LKB dose–response model based on the literature data from Burman et al. [16,24], concerning full organ irradiation ($V_{eff}=1$). The blue curves represent the adapted LKB dose–response model for esophagitis grade 1 and grade 2^+^. The dotted blue line corresponds to the irradiation of the lowest partial volume in the patient cohort. Grayed-out curves show the supposed course for higher dose values >50 Gy. Optimized model parameters were determined via LMQ optimization. The patient-specific NTCPs with individual irradiated effective volumes $V_{eff}$ are marked with a green X, which are summed up into groups (red squares).

**Adapted LKB model for the prediction of NTCP:** The adapted dose–response curves for the incidence of grade 1 and 2^+^ esophagitis are shown in Figure 1. The optimized parameters for the esophagitis grade 1 and 2^+^ were ${TD}_{50}=57.5$ Gy, m=0.55; and ${TD}_{50}=82.3$ Gy, m=0.4, respectively (Table 2). The fit quality for grade 1 and 2^+^ esophagitis according to the RSS, $R^{2}$, adjusted $R^{2}$, and Brier scores were satisfactory and within an acceptable range. Finally, it can be assumed that the adapted NTCP model can properly predict the probability of developing acute side effects for doses ≤ 50 Gy.

**Figure 2:**
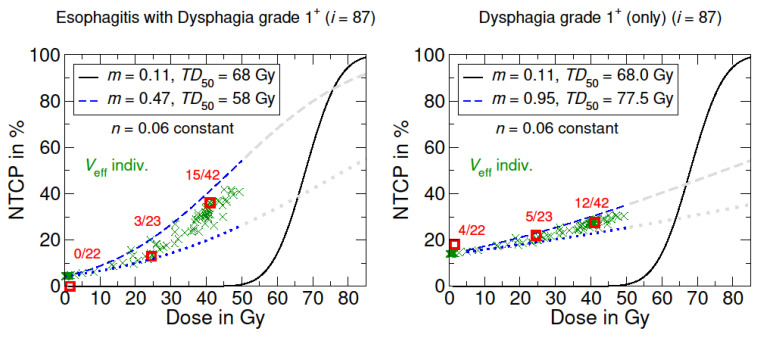
NTCP models for radiation-induced esophagitis with grade 1^+^ dysphagia and only dysphagia. The black curve shows the origin LKB dose–response model based on the literature data from Burman et al. [16,24], concerning full organ irradiation ($V_{eff}=1$). The blue curves represent the adapted LKB dose–response model. The dotted blue line corresponds to the irradiation of the lowest partial volume in the patient cohort. Grayed-out curves show the supposed course for higher dose values >50 Gy. Optimized model parameters were determined via LMQ optimization. The patient-specific NTCP with individual irradiated effective volumes $V_{eff}$ are marked with a green X, which are summed up into groups (red squares).

Concerning the adapted dose–response curve for esophagitis with grade 1^+^ dysphagia as shown in Figure 2 the characteristic sigmoidal dose–response curve is preserved; whereas, in contrast, the NTCP curve of dysphagia (only) showed an approximately linear dose–response relationship.

**Figure 3:**
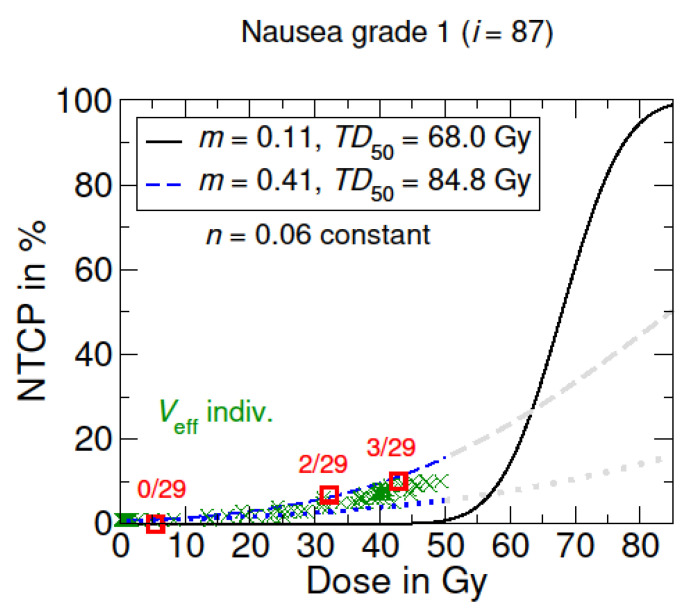
NTCP model for radiation-induced nausea. The black curve shows the origin LKB dose–response model based on the literature data from Burman et al. [16,24], concerning full organ irradiation ($V_{eff}=1$). The blue curves represent the adapted LKB dose–response model for a nausea grade 1. The dotted blue line corresponds to the irradiation of the lowest partial volume in the patient cohort. Grayed-out curves show the supposed course for higher dose values >50 Gy. Optimized model parameters were determined via LMQ optimization. The patient-specific NTCP with individual irradiated effective volumes $V_{eff}$ are marked with a green X, which are summed up into groups (red squares).

In Figure 3, the adapted NTCP model for nausea grade 1 exhibited the highest ${TD}_{50}=84.8$ compared to the acute toxicities of esophagitis and dysphagia. In addition, it shows high similarities to the NTCP curve of esophagitis grade 2^+^.

**The influence of reduced radiation doses on the NTCP of acute side effects:** The NTCP values for acute side effects were calculated using the adapted model with optimized model parameters. Comparisons were made between the “high dose” treatment plans and the treatment plans with “reduced doses”. The results demonstrated significant differences in mean NTCP values for specific side effects (α=0.05). As provided in Table 3, the NTCP data revealed that, especially in the a-NHL subgroup, reducing radiation doses from 40 Gy to 30 Gy led to a decrease in the occurrence of three acute side effects, especially esophagitis grade 1 (${\bar{NTCP}}_{40Gy}=17.4\%\pm 9.1\%$ vs. ${\bar{NTCP}}_{30Gy}=12.3\%\pm 5.8\%$), esophagitis grade 2^+^ (${\bar{NTCP}}_{40Gy}=5.4\%\pm 3.3\%$ vs. ${\bar{NTCP}}_{30Gy}=3.3\%\pm 1.8\%$), and nausea grade 1 (${\bar{NTCP}}_{40Gy}=5.5\%\pm 3.2\%$ vs. ${\bar{NTCP}}_{30Gy}=3.5\%\pm 1.8\%$), respectively. These differences led to a mean decrease in NTCP for esophagitis grade 1 ($\Delta {\bar{NTCP}}_{40vs.30Gy}=-5.1\%\pm 3.5\%$ with a max-to-min interval [−11%; 0]), grade 2^+^ ($\Delta {\bar{NTCP}}_{40vs.30Gy}=-2.1\%\pm 1.6\%$ with [−6%; 0%]), as well as nausea grade 1 ($\Delta {\bar{NTCP}}_{40vs.30Gy}=-3.5\%\pm 1.8\%$ with [−7%; −1%]).

In the i-NHL subgroup, the mean NTCP for the same acute side effects varied depending on radiation doses from 30 Gy to 24 Gy in a range of (${\bar{NTCP}}_{30Gy}=13.9\%\pm 11.1\%$ vs. ${\bar{NTCP}}_{24Gy}=9.1\%\pm 6.9\%$) for esophagitis grade 1, (${\bar{NTCP}}_{30Gy}=4.3\%\pm 4.3\%$ vs. ${\bar{NTCP}}_{24Gy}=2.3\%\pm 2.0\%$) for esophagitis grade 2^+^, and (${\bar{NTCP}}_{30Gy}=4.3\%\pm 4.2\%$ vs. ${\bar{NTCP}}_{24Gy}=2.5\%\pm 2.3\%$) for nausea grade 1, resulting in a mean NTCP decrease for esophagitis grade 1 ($\Delta {\bar{NTCP}}_{30vs.24Gy}=-3.0\%\pm 3.2\%$ with [−6%; 0]), grade 2^+^ ($\Delta {\bar{NTCP}}_{24vs.30Gy}=-1.3\%\pm 1.6\%$ with [−6%; 0]), and nausea grade 1 ($\Delta {\bar{NTCP}}_{30vs.24Gy}=-1.0\%\pm 1.3\%$ with [−6%; 0]). 

Regarding radiation-induced pneumonitis, pericarditis, or myelitis, no significant differences in NTCP were observed for doses in the range of from 40 Gy to 24 Gy. The comprehensive calculated NTCP are provided in Table 3. 

## 4. Discussion

The primary concern in modern radiation therapy for improving the outcomes of lymphoma patients lies in minimizing acute to long-term side effects. Enhanced diagnostic imaging, immobilization techniques, and improved image guidance during radiation therapy facilitate a more precise target margin, ensuring effective treatment while minimizing exposure to adjacent normal tissues, thereby mitigating the potential for long-term toxicity. Specifically, IMRT or PBT demonstrates significant efficacy in reducing doses to certain organs at risk. In addition, Xiang et al. revealed that the risk of a secondary malignancies diagnosis after IMRT was comparable to 3D conformal radiation therapy, whereas PBT was associated with a potentially lower risk [38]. The absolute incidence of secondary cancer is relatively low (approximately 1.5 per 100 person years) but nonetheless crucial to minimize [38]. In this study, the adoption of involved site radiation therapy (ISRT) in IMRT or PBT was aimed to minimize treatment-related toxicities while ensuring adequate target coverage. However, it is imperative that radiation therapy planning and the choice of irradiation technique be individually tailored for each patient through the comparison of multiple treatment options [2,22,39,40,41]. 

Following Reinartz et al. and Eich et al., who used the LKB dose–response model to investigate the effects of field size and dose dependencies on normal tissue toxicity, this study primarily focuses on the adaption of the LKB model to adequately describe the occurrence of acute and intermediate-term side effects in the treatment of NHL, particularly of the neck/thorax region [6,23]. The optimized LKB model parameters $TD_{50}$ and *m* in this study made it possible to predict the probabilities of acute side effects accurately in comparison to the observed NTCP in the patient cohort. The organ-specific volume dependence parameter *n* remains thereby constant. Qualitatively, the adjusted NTCP model for grade 1^+^ dysphagia (only) is approximately linear in the dose range ≤ 50 Gy, whereby all adjusted models show, in common, that even small radiation doses can lead to a non-negligible risk of acute side effects. These findings indicate that dose–response relationships for selected acute side effects may differ strongly from the models for late side effects described in the literature by Burman et al. [36] (Figure 2).

Nevertheless, some limitations should be considered. At first, increasing the patient cohort might further improve the accuracy of the predicting NTCP model. Secondly, toxicities in this study are only reported for the entire treatment series without specifying the time of onset during the treatment. In future analyses, it would be useful to specify the toxicity at the time of first occurrence in the radiation series in order to further improve the NTCP model. Furthermore, it is important to emphasize that the planned dose is never exactly the applied dose. The difference depends inter alia on planning technique, imaging frequency, anatomy fluctuations, and patient compliance. The use of image-guided or daily adapted radiotherapy techniques can improve the geometric accuracy and precision of radiation delivery and thus help to reduce the deviations between planned and applied dose [42,43]. Another factor that influences the quality of data included in the NTCP model adaption might be the interobserver variability among different clinicians for head and neck cancer patients. As reported by Kalendralis and Sloep et al., these variabilities can directly influence the performance of the adapted NTCP model in different independent patient cohorts [44]. It is also worth noting that in a related study, the biophysical model displayed sensitivity to parameter variations, where even a 1% deviation in $TD_{50}$ resulted in a substantial 10% deviation in the NTCP [6,23]. It is also prudent to validate if the adapted model’s predictions are accurate and transferable before using it for other patient cohorts [22]. Nevertheless, the feasibility of biophysical models to estimate the risk of normal tissue toxicity and its ability to provide valuable information to guide treatment planning decisions was extensively confirmed in the literature [3,6,13,15,17,45].

Moreover, the results demonstrated that a combined NTCP model for esophagitis with dysphagia is more appropriate compared to a separate view of the symptom only. Thus, it could be questionable to investigate the symptom of dysphagia separately from its causal effect or diagnosis. In addition, some patients already have dysphagia at the beginning of treatment, e.g., caused by chemotherapy or by the NHL itself. This is also reflected in the NTCP curve, since at 0 Gy there is already a probability of esophagitis of approximately 5% or 12% for dysphagia (Figure 1 and Figure 2). In the context of acute symptoms, which occur during radiation therapy, relating them to a DVH representing the entire treatment course might be problematical. Instead, Marks et al. suggested to link acute events to the dose administered before symptom onset, especially considering any latency time [22]. This procedure could be a better way to derive a more accurate dose–response model for the onset of acute symptoms. 

The analysis of the implications of reduced radiation doses revealed that the probability of developing acute side effects, such as esophagitis grade 1 or higher (as one of the causal causes of nausea or dysphagia), can be reduced by up to 11% (Table 3). Several studies have shown that higher radiation doses are associated with increased toxicity, emphasizing the importance of optimizing radiation protocols to spare normal tissues [13,14,15,22,23]. For example, Granton et al. investigated the effects of reduced radiation doses and the use of IMRT on normal tissue toxicity in thoracic radiation [20]. Their findings demonstrated a significant decrease in acute side effects, including pneumonitis and dysphagia, with the use of IMRT. This supports the results of this analysis, indicating that both subgroups of lymphoma patients are less likely to develop acute esophageal adverse side effects when treated with reduced radiation doses. In addition, the presented findings are also in conjunction with the existing literature and underline the feasibility and potential benefits of reducing radiation doses in NHL management [9,13,14,15,17,22,25,46]. 

While this study supports the feasibility of reduced radiation doses in NHL management, it is important to acknowledge that firstly, to validate the findings and comprehensively assess the long-term impact of reduced radiation doses, larger prospective studies with extended follow-up periods are imperative. Studies resembling the scope of Eich et al., involving larger patient cohorts and prolonged observation, are crucial in this context [23]. Secondly, findings in this study primarily pertain to the short- to intermediate-term complications, whereas the long-term implications of reduced radiation doses, particularly regarding late side effects, remain to be comprehensively evaluated. Moreover, it is important to acknowledge the inherent difficulty in distinguishing between pneumonitis and pneumonia due to the limited routine use of imaging, which may have impacted the accurate assessment of pneumonitis incidence and severity. As highlighted in a related study by Oertel et al., the occurrence of pulmonary toxicities, including pneumonia and pneumonitis, can present challenges in clinical diagnosis [47]. The present study shares a similar complexity in distinguishing between these pulmonary conditions, which underscores the importance of further research and multidisciplinary approaches to enhance accuracy in clinical assessments. Additionally, the heterogeneity in NHL subtypes and treatment regimens within the patient cohort may have influenced the observed treatment outcomes. However, with the help of robust normal tissue toxicity models and by implementing reduced prescribed doses, radiation oncologists attempt to achieve a balance between disease control and the occurrence of treatment-related side effects [9,22,46].

## 5. Conclusions

Clinical DVH data and toxicity outcomes after irradiation of non-Hodgkin lymphoma (NHL) in the neck/thorax region were analysed and embedded into an adapted NTCP model which can properly predict the occurrence of different acute side effects for doses ≤ 50 Gy in the patient cohort. It was shown that the probability of developing acute side effects can deviate considerably from the predicted NTCP derived from commonly used NTCP models in the literature. Additionally, the adapted NTCP models offered quantitative insights into the potential benefits of utilizing reduced radiation doses: it could be demonstrated that lower prescribed doses in the treatment of NHL in the neck/thorax region can minimize the NTCP of different acute side effects significantly. For example, a decrease of 20 to 25% in prescribed dose can reduce the probability of developing acute side effects such as esophagitis grade 1 by up to 11% for aggressive NHL subtypes and 6% for indolent NHL, respectively. More detailed knowledge about the relationship between dose and the occurrence of acute side effects might also be helpful to optimize dose prescriptions and treatment plans with the overall goal of increasing the treatment quality and, especially, the outcomes for patients. Therefore, it is necessary to further improve existing NTCP models and to investigate the impact of modern reduced radiation doses on both acute and late side effects.

## Figures and Tables

**Table 1:** Overview of patient characteristics (age, histopathological aggressiveness of NHL, and tumor localization) and the number of acute side effects observed in the patient cohort.

					In Total/*i*
Age (year)					
Mean	59				
Range	19–88				
Non-Hodgkin lymphoma	aggressive	indolent			
	68 (60%)	46 (40%)			114
Localization					
Nodal	cervical	mediastinal	axillary		
	44 (38%)	32 (7%)	8 (28%)		84
Extranodal	thoracic	cervical spine	breast	parotid gland	
	22 (19%)	2 (2%)	7 (6%)	1 (1%)	32
Esophagitis	grade 1	grade 2	grade 3		
	14 (16%)	4 (5%)	1 (1%)		19/87
with Dysphagia	grade 1	grade 2	grade 3		
	12 (14%)	5 (6%)	1 (1%)		18/87
only Dysphagia	grade 1	grade 2			
	18 (21%)	3 (3%)			21/87
Nausea	grade 1				
	5 (6%)				5/87
Pneumonitis	grade 1				
	2 (3%)				2/67
Pericarditis	grade 1				
	1 (1%)				1/77
Myelitis	grade 1				
	2 (2%)				2/88

Note: *i* corresponds to the number of available patient dose–volume histogram (DVH).

**Table 2:** NTCP LKB parameters for acute side effects and the quality of fit analysis. Note that NTCP model parameters for pneumonitis grade 1, pericarditis grade 1, and myelitis grade 1 were taken from [24].

Side Effect	$TD_{50}$	m	RSS	$R^{2}$	$\mathrm{Adjusted}\;R^{2}$	Brier Score	Brier Skill Score	AIC/i
Esophagitis								
grade 1	57.5	0.55	0.119	0.826	0.822	0.143	0.114	−3.13
grade 2^+^	82.3	0.40	0.019	0.761	0.755	0.043	0.066	−5.45
with Dysphagia								
grade 1^+^	58.0	0.47	0.256	0.812	0.806	0.141	0.317	−2.34
only Dysphagia								
grade 1^+^	77.5	0.95	0.117	0.589	0.575	0.190	0.249	−3.87
Nausea								
grade 1	84.8	0.41	0.037	0.531	0.520	0.056	0.015	−5.18
Pneumonitis								
grade 1	24.5	0.18	-	-	-	-	-	-
Pericarditis								
grade 1	48.0	0.36	-	-	-	-	-	-
Myelitis								
grade 1	66.5	0.17	-	-	-	-	-	-

Abbreviations: $TD_{50}\;\mathrm{and}\;m$ are specific model parameters from the LKB model; *RSS* corresponds to the residual sum of squares; $R^{2}$ is the sum of squared residuals, calculated as $R^{2}=1-RSS/TSS$, where *TSS* denotes the total sum of squares, and AIC/i is the Akaike information criterion divided by the number of data points *i*.

**Table 3:** LKB calculated NTCP for different side effects depending on the prescribed dose and the aggressiveness of NHL. ${\bar{NTCP}}_{xGy}$ corresponds to the mean NTCP in the patient cohort irradiated with the prescribed dose *x* and $\Delta {\bar{NTCP}}_{x_{1}vs.x_{2}Gy}$ is the mean decrease in NTCP, whereby the column max-to-min is the associated maximum and minimum decrease in NTCP after reducing the prescribed dose from $x_{1}$ to $x_{2}$.

Side Effect	a-NHL (*i* = 68)				i-NHL (*i* = 46)			
	${\bar{NTCP}}_{40Gy}$	${\bar{NTCP}}_{30Gy}$	$\Delta {\bar{NTCP}}_{40vs.30Gy}$	max-to-min	${\bar{NTCP}}_{30Gy}$	${\bar{NTCP}}_{24Gy}$	$\Delta {\bar{NTCP}}_{30vs.24Gy}$	max-to-min
Esophagitis								
grade 1	17.4% ± 9.1%	12.3% ± 5.8%	−5.1% ± 3.5%	[−11%; 0%]	13.9% ± 11.1%	9.1% ± 6.9%	−3.0% ± 3.4%	[−11%; 0%]
grade 2^+^	5.4% ± 3.3%	3.3% ± 1.8%	−2.1% ± 1.6%	[−6%; 0%]	4.3% ± 4.3%	2.3% ± 2.0%	−1.3% ± 1.6%	[−6%; 0%]
with Dysphagia								
grade 1^+^	23.8% ± 13.0%	16.8% ± 8.3%	−6.9% ± 4.8%	[−15%; 0%]	18.9% ±15.7%	12.3% ± 9.6%	−3.9% ± 4.3%	[−14%; 0%]
only Dysphagia								
grade 1^+^	26.2% ± 6.4%	20.9% ± 4.8%	−5.3% ± 1.6%	[−8%; −3%]	23.8% ± 7.2%	20.8% ± 4.9%	−1.7% ± 1.6%	[−5%; 0%]
Nausea								
grade 1	5.5% ± 3.2%	3.5% ± 1.8%	−3.5% ± 1.8%	[−7%; −1%]	4.3% ± 4.2%	2.5% ± 2.3%	−1.0% ± 1.3%	[−6%; 0%]
Pneumonitis								
grade 1	<1%	<1%	<1%	[−0.7%; 0%]	<1%	<1%	ND	ND
Pericarditis								
grade 1	0%	0%	ND	ND	0%	0%	ND	ND
Myelitis								
grade 1	<1%	<1%	<1%	[−0.7%; 0%]	<1%	<1%	ND	ND

Abbreviations: ND—no difference.

## Data Availability

The data presented in this study are available in the article.
